# Higher yields of hybrid rice do not depend on nitrogen fertilization under moderate to high soil fertility conditions

**DOI:** 10.1186/s12284-017-0182-1

**Published:** 2017-09-21

**Authors:** Min Huang, Peng Jiang, Shuanglü Shan, Wei Gao, Guohui Ma, Yingbin Zou, Norman Uphoff, Longping Yuan

**Affiliations:** 1 Southern Regional Collaborative Innovation Center for Grain and Oil Crops (CICGO), Hunan Agricultural University, Changsha, 410128 China; 2 International Programs-College of Agriculture and Life Sciences (IP-CALS), Cornell University, Ithaca, 14853 USA; 3 State Key Laboratory of Hybrid Rice, China National Hybrid Rice Research and Development Center, Changsha, 410125 China

**Keywords:** Grain yield, Hybrid rice, Nitrogen inputs, Sustainable crop production

## Abstract

**Background:**

Increasing rice yield with fewer external inputs is critical to ensuring food security, reducing environmental costs, and improving returns. Use of hybrid rice has expanded greatly in China due to its higher yield potential. Meanwhile, large and increasing amounts of nitrogen (N) fertilizers have been used for expanding rice production in China. It is not clear to what extent the success of hybrid rice in China is associated with N fertilizer inputs.

**Findings:**

We observed that the higher grain yield with N fertilizer in hybrid rice was driven more by a higher yield without N fertilizer than by increases in grain yield with N fertilizer under moderate to high soil fertility conditions.

**Conclusions:**

Our results suggest that greater application of N fertilizers is not needed to benefit from hybrid rice production under moderate to high soil fertility conditions, and that improving and maintaining soil fertility should be a focus for sustaining hybrid rice production. Moreover, our study also indicates that zero-N testing may be a potentially useful tool to develop hybrid rice with high yield and without requirement of greater external N inputs under moderate to high soil fertility conditions.

**Electronic supplementary material:**

The online version of this article (10.1186/s12284-017-0182-1) contains supplementary material, which is available to authorized users.

## Findings

Rice is a staple food for over half of the world’s population (Muthayya et al. 2014). Rice yield must increase by at least 1% annually to meet the growing demand for food that will result from population growth and economic development (Normile 2010). Rice cultivars with higher yield potential are essential to achieve this goal (Peng et al. 2008). Moreover, it is also important to minimize the dependence on external inputs for crop production to reduce adverse environmental impacts, and to get the greatest possible expression of crops’ yield potential.

Over the past three decades, the use of large amounts of external inputs such as inorganic fertilizers, especially nitrogen (N), has imposed substantial environmental costs, including increased greenhouse gas emissions (Davidson 2009), enhanced N deposition (Liu et al. 2013), soil acidification (Guo et al. 2010), surface water eutrophication (Le et al. 2010), and biodiversity loss (Christopher and Tilman 2008). Diminishing returns are being observed with the use of such fertilizers. In China since the start of its Green Revolution, the ratio of incremental increases in rice production in response to additional applications of N fertilizer has fallen sharply (Peng et al. 2010).

The development of hybrid cultivars has made a major contribution to increasing the yield potential of rice crops (Yuan 1994). Hybrid rice cultivars have a yield advantage of about 10–20% over improved inbred rice cultivars (Peng et al. 1999; Cheng et al. 2007). China is the first country to exploit the potentials of hybrid rice commercially on a large scale (Cheng et al. 2004). The area planted under hybrid rice there has expanded greatly since the late 1970s, accounting for more than half of the total national rice-growing area in recent years (Yuan 2014).

At the same time, fertilizer consumption, especially N, has increased almost linearly in China (Fan et al. 2011). At present, China with approximately 20% of the world’s rice-growing area consumes nearly 40% of the total N fertilizer used for rice production (Yan et al. 2006). The average N application rate for rice production in China is 180 kg ha^−1^, about 75% higher than the world average (Peng et al. 2002; Chen et al. 2014). Moreover, the high yields of hybrid rice cultivars are often achieved when large amounts of resources (including N fertilizers) are provided, which leads to a perception that hybrid rice cultivars perform better than inbred rice cultivars only under high-input conditions (Yuan et al. 2017). These raise the question of whether the success of hybrid rice in China is related with N fertilizer inputs. Does the higher yield from hybrid rice depend on N fertilization? This is a common concern for farmers who wish to begin planting hybrid rice.

To address the question, two field experiments were conducted during 2012 to 2016 (Additional file 1: Materials and Methods). In each experiment, the treatments included a zero-N control and two rates of N fertilization, moderate and high. In one field experiment (Experiment I, 2012–2014), we compared grain yield with N fertilizer (Y_N_) with the grain yield obtained without N fertilizer (Y_0_), also comparing differences between hybrid and inbred rice cultivars in terms of the increases in grain yield attained with N fertilizer (∆Y_N_). Then, in another field experiment (Experiment II, 2015–2016), we evaluated the trends in Y_N_, Y_0_ and ∆Y_N_ in representative hybrid rice cultivars selected to represent different phases of hybrid development since 1996.

Because the interaction effect between cultivar and N rate on grain yield was not significant in all five experiments (Additional file 2: Table S1), in the figures below we report the data on yield and yield increases as means for the two N fertilizer rates, making interpretation easier.

**Fig. 1:**
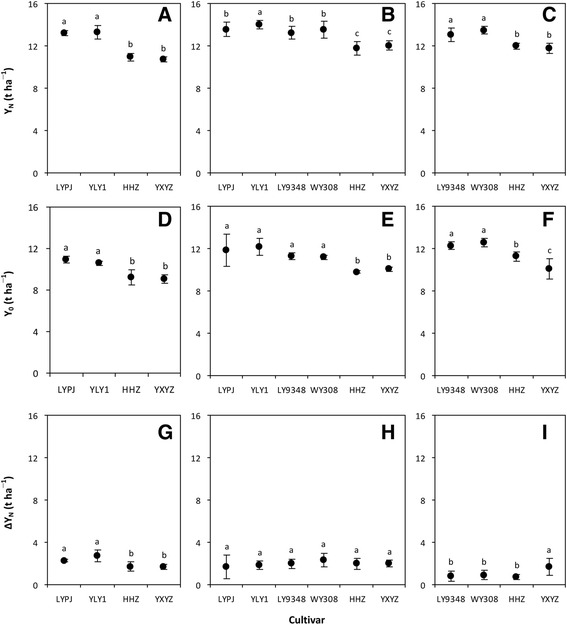
Grain yield with N fertilizer (Y_N_, **a**–**c**), grain yield without N fertilizer (Y_0_, **d**–**f**), and increase in grain yield with N fertilizer (∆Y_N_, **g**–**i**) of hybrid and inbred rice cultivars grown in Xingyi, Guizhou Province, China in 2012 (**a**, **d** and **g**), 2013 (**b**, **e** and **h**) and 2014 (**c**, **f** and **i**). LYPJ (Liangyoupeijiu), YLY1 (Y-liangyou 1), LY9348 (Luoyou 9348) and WY308 (Wuyou 308) are hybrid rice cultivars. HHZ (Huanghuazhan) and YXYZ (Yuxiangyouzhan) are inbred rice cultivars. Data in **a**–**c** and **g**–**i** are the means across two N fertilizer rates. Data points are means and standard deviations of three replications (**d**–**f**) or six replications (**a**–**c** and **g**–**i**). Data points marked with the same letters are not significantly different at the 0.05 probability level according to LSD test

In the Experiment I, the average Y_N_ for the hybrid cultivars Liangyoupeijiu (LYPJ) and Y-liangyou 1 (YLY1) was 22% and 16% higher than the Y_N_ for the inbred cultivars Huanghuangzhan (HHZ) and Yuxiangyouzhan (YXYZ) in 2012 and 2013, respectively (Fig. 1a and b). Then in 2013 and 2014, the hybrid cultivars Luoyou 9348 (LY9348) and Wuyou 308 (WY308) had about a 12% higher average Y_N_ than did the inbred cultivars HHZ and YXYZ (Fig. 1b and c). These magnitudes of yield advantage with hybrid compared to inbred cultivars are similar to those reported in previous studies (Peng et al. 1999; Cheng et al. 2007).

More interestingly, our results showed that the average Y_0_ from hybrid cultivars LYPJ and YLY1 was 18% and 21% higher in 2012 and 2013, respectively, than from inbred cultivars HHZ and YXYZ (Fig. 1d and e). Hybrid cultivars LY9348 and WY308 had 13% and 16% higher average Y_0_ than did the inbred cultivars HHZ and YXYZ in 2013 and 2014, respectively (Fig. 1e and f). However, the difference in ∆Y_N_ between hybrid and inbred cultivars was not consistent or statistically significant (Fig. 1g–i).

These results indicate that the higher Y_N_ in hybrid compared with inbred rice cultivars was driven more by their higher Y_0_ than by ∆Y_N_, which suggests that the higher yield of hybrid rice does not depend on N fertilization under the experimental soil conditions. This might be attributed to the fact that hybrid rice plants generally have larger, deeper, and more vigorous root systems (Yang and Sun 1986; Zhang et al. 2009), and consequently they are able to absorb more indigenous soil N than inbred rice plants can.

Consistently, in a micro-plot experiment with application of ^15^N labeled urea (Additional file 1: Materials and Methods), we observed that the hybrid rice cultivars LYPJ and YLY1 had significantly more indigenous soil N uptake, but equal labeled-N uptake, compared to the inbred rice cultivars HHZ and YXYZ (Additional file 3: Table S2). These differences highlight the need for greater fundamental understanding of roots’ morphology and physiology in relation to the greater uptake of indigenous soil N in hybrid rice.

**Fig. 2:**
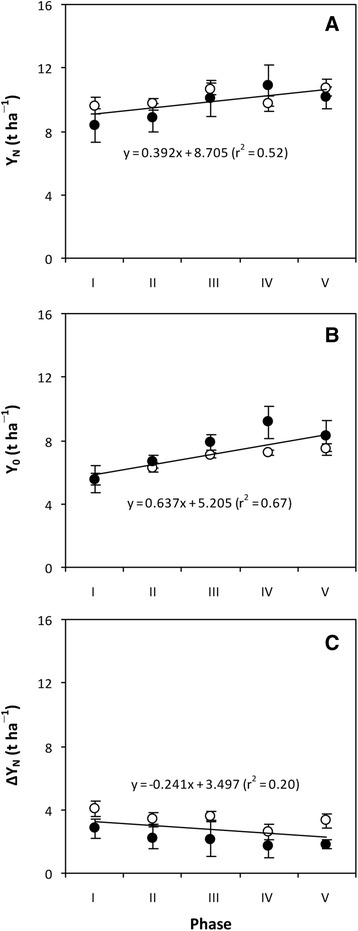
Trends in grain yield with N fertilizer (Y_N_, **a**), grain yield without N fertilizer (Y_0_, **b**), and increase in grain yield with N fertilizer (∆Y_N_, **c**) of representative hybrid rice cultivars developed during different phases in China since 1996. Phases I, II, III, IV and V are 1996–2000, 2001–2005, 2006–2010, 2011–2015, and 2016–, respectively. Data were obtained from field experiments in which five representative hybrid rice cultivars of the five phases (i.e., Liangyoupeijiu, Y-liangyou 1, Y-liangyou 2, Y-liangyou 900, and Chaoyou 1000) were grown in Ningxiang, Hunan Province, China in 2015 (open circle) and 2016 (closed circle). Data in **a** and **c** are the means across two N fertilizer rates. Data points are means and standard deviations of three replications (**b**) or six replications (**a** and **c**). Trend (slope) in **c** is not statistically significant at the 0.05 probability level according to Student’s t test

In the Experiment II, both Y_N_ and Y_0_ were significantly increased while no significant trend was observed for ∆Y_N_ in representative hybrid rice cultivars developed during different phases since 1996 (Fig. 2a–c). These results indicate that root system may be improved with the development of new hybrid rice cultivars, and also support the inference that the higher yield of hybrid rice does not depend on N fertilization.

**Fig. 3:**
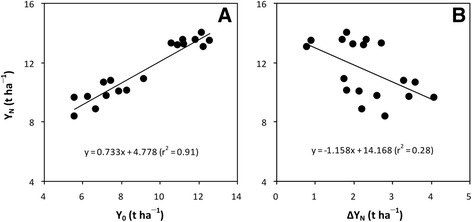
Relationships of grain yield with N fertilizer (Y_N_) to grain yield without N fertilizer (Y_0_, **a**) and increase in grain yield with N fertilizer (∆Y_N_, **b**) in hybrid rice cultivars. Data are a combination of those from hybrid rice cultivars in Figs. 1 and 2

In addition, our results showed that there was a large difference in Y_N_ in the hybrid rice cultivars LYPJ and YLY1 between the two experiments (Fig. 1a and b; Fig. 2a). The average Y_N_ from LYPJ and YLY1 was 48% higher in the Experiment I than in the Experiment II. The yield gap in Y_N_ observed between the two experiments was mainly attributable to the difference in Y_0_ (Fig. 1d and e; Fig. 2b) because the difference in ∆Y_N_ was contrary to that in Y_N_ (Fig. 1h and i; Fig. 2c). By combining the data of hybrid rice cultivars in the Experiment I and II, it was observed that Y_N_ was positively correlated with Y_0_ but negatively with ∆Y_N_ (Fig. 3a and b). Such results again support the conclusion that the higher yield of hybrid rice does not depend on N fertilization.

Because our experiments were conducted under moderate to high soil fertility conditions (Additional file 1: Materials and Methods), the results have implications for the further production and development of hybrid rice under such conditions. First, the higher Y_N_ in hybrid rice cultivars was driven more by higher Y_0_ than by ∆Y_N_, and therefore greater use of N fertilizers is not needed for more hybrid rice production so much as making improvements in soil fertility should be the focus for sustained hybrid rice production. Second, we note that a super-high Y_N_ of more than 13 t ha^−1^ was obtained from hybrid rice in the Experiment I (Fig. 1a–c), with about 85% of such super-high Y_N_ contributed by Y_0_ (Fig. 1d–f). These results indicate that zero-N testing may be a potentially useful tool to develop super high-yielding hybrid rice without requirement of more external N inputs.

## Additional files

Additional file 1: Materials and Methods. (DOC 54 kb)

Additional file 2: Table S1.Grain yield in rice cultivars grown under two N fertilizer rates in Xingyi, Guizhou Province, China in 2012–2014 (Experiment I), and in Ningxiang, Hunan Province, China in 2015 and 2016 (Experiment II). (DOC 53 kb)

Additional file 3: Table S2.Indigenous soil N uptake and labeled-N uptake in rice cultivars in a micro-plot experiment with application of 15 N labeled urea in Changsha, Hunan Province in China in 2013. (DOC 31 kb)

## Abbreviations

∆Y_N_: Increases in grain yield attained with N fertilizer
HHZ: Rice cultivar Huanghuangzhan
LY9348: Rice cultivar Luoyou 9348
LYPJ: Rice cultivar Liangyoupeijiu
N: Nitrogen
WY308: Rice cultivar Wuyou 308
Y_0_: Grain yield obtained without N fertilizer
YLY1: Rice cultivar Y-liangyou 1
Y_N_: Grain yield obtained with N fertilizer
YXYZ: Rice cultivar Yuxiangyouzhan
