# Supplementary material for: Higher yields of hybrid rice do not depend on nitrogen fertilization under moderate to high soil fertility conditions
Source: Rice (N Y). 2017 Sep 21;10:43. doi: 10.1186/s12284-017-0182-1 (PMC5608657; doi:10.1186/s12284-017-0182-1)
Supplement: Supplementary file 1 — Materials and Methods. (DOC 54 kb) [file 12284_2017_182_MOESM1_ESM.doc]

**Materials and Methods**

**Field experiments**

Field experiments were conducted in Xingyi, Guizhou Province, China during 2012 to 2014 (Experiment I) and in Ningxiang, Hunan Province, China in 2015 and 2016 (Experiment II). The soil of the Xingyi site had the following properties: pH 7.9, 52.6 g kg^−1^ organic matter, 2.6 g kg^−1^ total N, 16.5 mg kg^−1^ available P, and 257 mg kg^−1^ available K. The soil of the Ningxiang site had the following properties: pH 5.8, 30.6 g kg^−1^ organic matter, 1.4 g kg^−1^ total N, 27.6 mg kg^−1^ available P, and 85.8 mg kg^−1^ available K.

In the Experiment I, two hybrid rice cultivars, Liangyoupeijiu (LYPJ) and Y-liangyou 1 (YLY1), and two inbred cultivars, Huanghuazhan (HHZ) and Yuxiangyouzhan (YXYZ), were used in 2012. Four hybrid rice cultivars, LYPJ, YLY1, Luoyou 9348 (LYP348) and Wuyou 308 (WY308), and two inbred rice cultivars, HHZ and YXYZ, were used in 2013. Two hybrid rice cultivars, LY9348 and WY308, and two inbred rice cultivars, HHZ and YXYZ, were used in 2014. These cultivars have been widely grown by rice farmers in China because of their high yields. In the Experiment II, five hybrid rice cultivars, LYPJ, YLY1, Y-liangyou 2, Y-liangyou 900 and Chaoyou 1000, were used in 2015 and 2016. These cultivars are recommended by the China National Hybrid Rice Research and Development Center as representative hybrid rice cultivars for the 1996–2000, 2001–2005, 2006–2010, 2011–2015, and 2016– periods of hybrid rice development in China.

Treatments were arranged in a split-plot design with N rates as the main plots with cultivars as subplots. The experiment was replicated three times. In the Experiment I, the N rates were 0 kg N ha^−1^ (zero-N control), 161 kg N ha^−1^ (moderate N) and 225 kg N ha^−1^ (high N). With the moderate N rate, 56, 60, 45 and 0 kg N ha^−1^ were applied at basal (1 day before transplanting), mid-tillering, panicle initiation and booting, respectively. With the high N rate, 112.5, 45, 45 and 22.5 kg N ha^−1^ were applied at basal, mid-tillering, panicle initiation and booting, respectively. In the Experiment II, the N rates were 0 kg N ha^−1^ (zero-N control), 210 kg N ha^−1^ (moderate N) and 300 kg N ha^−1^ (high N). With the moderate N rate, 105, 42 and 63 kg N ha^−1^ were applied at basal, mid-tillering, and panicle initiation, respectively. With the high N rate, 150, 60 and 90 kg N ha^−1^ were applied at basal, mid-tillering and panicle initiation, respectively.

Pre-germinated seeds were sown in seedbeds. In the Experiment I, 35-day-old seedlings were transplanted at a hill spacing of 20 cm × 27 cm with two seedlings per hill. Phosphorus (112.5 kg P_2_O_5_ ha^−1^) was applied and incorporated in all subplots as basal. Potassium (157.5 kg K_2_O ha^−1^) was split equally at basal and panicle initiation. In the Experiment II, 25-day-old seedlings were transplanted at a hill spacing of 30 cm × 20 cm with two seedlings per hill. Phosphorus was applied and incorporated in all subplots as basal, 105 and 150 kg P_2_O_5_ ha^−1^ for the moderate and high N treatments, respectively. Potassium was split equally at basal and panicle initiation, 210 and 300 kg K_2_O ha^−1^ for the moderate and high N treatments, respectively. The strategy for water management was in the sequence of flooding, midseason drainage, re-flooding, and moist intermittent irrigation. Weeds, insects and diseases were intensively controlled with chemicals to avoid yield loss.

Grain yield was determined from a 5-m^2^ area in the middle of each subplot at maturity and adjusted to a moisture content of 0.14 g H_2_O g^−1^ fresh weight.

**Micro-plot experiment**

A ^15^N-tracer micro-plot experiment was carried out in Changsha, Hunan Province, China in 2013. The micro-plots (PVC cylinders, 40-cm long with 40-cm inner diameter) were inserted into the soil to a depth of 20 cm with a collar of 20 cm aboveground. The soil had the following properties: pH 6.1, 24.9 g kg^−1^ organic matter, 1.4 g kg^−1^ total N, 38.3 mg kg^−1^ available P, and 72.9 mg kg^−1^ available K.

Two hybrid rice cultivars, LYPJ and YLY1, and two inbred cultivars, HHZ and YXYZ, were arranged in a randomized complete block design with six replications (micro-plots). Pre-germinated seeds were sown in seedbeds. Twenty five-day-old seedlings were transplanted at the rate of two seedlings per hill and three hills per micro-plot. Plants received 15 g N m^−2^ as ^15^N-labeled urea (abundance 5.18 atom%, producing by the Shanghai Institute of Chemical Industry, China), which was applied in three splits: 7.5 g N m^−2^ as basal (1 day before transplanting), 3.0 g N m^−2^ at mid-tillering, and 4.5 g N m^−2^ at panicle initiation. Phosphorus (7.5 g P_2_O_5_ m^−2^) was applied and incorporated in all micro-plots as basal. Potassium (15 g K_2_O m^−2^) was split equally at basal and panicle initiation. The strategy for water management was in the sequence of flooding, midseason drainage, re-flooding, and moist intermittent irrigation. Weeds, insects and diseases were intensively controlled with chemicals to avoid yield loss.

Aboveground plant parts in each micro-plot were sampled at maturity and oven-dried at 70°C to constant weight to determine total dry weight. The dried samples were ground to pass a 100-mesh sieve and a sub-sample of 0.20 g was digested with H_2_SO_4_-H_2_O_2_. Total N content (VAP50 Kjeldahl meter, Gerhardt, Königswinter, Germany) and ^15^N abundance (Delta V Advantage isotope mass spectrometer, Thermo Fisher, Waltham, MA, USA) were determined to calculate indigenous soil N uptake and labeled-N uptake according to the method of Huang et al (2014).

**Statistical analysis**

Data were analyzed using Statistix 8.0 (Analytical Software, Tallahassee, FL, USA). In the Experiment I, grain yield with N fertilizer (Y_N_), grain yield obtained without N fertilizer (Y_0_), and increase in grain yield attained with N fertilizer (∆Y_N_) were compared among cultivars using analysis of variance and followed by LSD test at the 0.05 probability level. In the Experiment II, the significance of trends in Y_N_, Y_0_ and ∆Y_N_ of the representative hybrid rice cultivars in different phases was determined by testing the statistical significance of slopes at the 0.05 probability level according to the Student’s *t* test. In addition, the data of hybrid rice cultivars in the Experiment I and II were combined to evaluate the relationships of Y_N_ to Y_0_ and ∆Y_N_ by using correlation analysis. In the micro-plot experiment, indigenous soil N uptake and labeled-N uptake were compared among cultivars using analysis of variance and followed by LSD test at the 0.05 probability level.

**References**

Huang M, Yang L, Qin H, Jiang L, Zou Y (2014) Fertilizer nitrogen uptake by rice increased by biochar application. Biol Fertil Soils 50:997–1000.
