# Supplementary material for: Higher yields of hybrid rice do not depend on nitrogen fertilization under moderate to high soil fertility conditions
Source: Rice (N Y). 2017 Sep 21;10:43. doi: 10.1186/s12284-017-0182-1 (PMC5608657; doi:10.1186/s12284-017-0182-1)
Supplement: Supplementary file 2 — Table S1.Grain yield in rice cultivars grown under two N fertilizer rates in Xingyi, Guizhou Province, China in 2012–2014 (Experiment I), and in Ningxiang, Hunan Province, China in 2015 and 2016 (Experiment II). (DOC 53 kb) [file 12284_2017_182_MOESM2_ESM.doc]

**Table S1.** Grain yield (t ha^−1^) in rice cultivars grown under two N fertilizer rates in Xingyi, Guizhou Province, China in 2012–2014 (Experiment I), and in Ningxiang, Hunan Province, China in 2015 and 2016 (Experiment II)^a^

| Cultivar^b^ | 2012 | |  | 2013 | |  | 2014 | |  | 2015 | |  | 2016 | |
| --- | --- | --- | --- | --- | --- | --- | --- | --- | --- | --- | --- | --- | --- | --- |
|  | Moderate N | High N |  | Moderate N | High N |  | Moderate N | High N |  | Moderate N | High N |  | Moderate N | High N |
| LYPJ | 13.20 | 13.18 |  | 13.88 | 13.21 |  | − | − |  | 9.21 | 10.02 |  | 7.99 | 8.75 |
| YLY1 | 13.68 | 12.91 |  | 13.90 | 14.09 |  | − | − |  | 9.41 | 10.00 |  | 8.44 | 9.32 |
| LY9348 | − | − |  | 13.40 | 13.07 |  | 12.79 | 13.33 |  | − | − |  | − | − |
| WY308 | − | − |  | 13.67 | 13.37 |  | 13.19 | 13.75 |  | − | − |  | − | − |
| HHZ | 11.08 | 10.77 |  | 11.77 | 11.74 |  | 12.15 | 11.82 |  | − | − |  | − | − |
| YXYZ | 10.78 | 10.68 |  | 12.13 | 11.95 |  | 12.13 | 11.41 |  | − | − |  | − | − |
| YLY2 | − | − |  | − | − |  | − | − |  | 10.63 | 10.71 |  | 9.11 | 10.97 |
| YLY900 | − | − |  | − | − |  | − | − |  | 9.45 | 10.09 |  | 10.30 | 11.50 |
| CY1000 | − | − |  | − | − |  | − | − |  | 10.36 | 11.15 |  | 9.98 | 10.25 |
| Analysis of variance |  |  |  |  |  |  |  |  |  |  |  |  |  |  |
| Cultivar (C) | ** |  |  | ** |  |  | ** |  |  | ** |  |  | ** |  |
| N rate (N) | ** |  |  | * |  |  | NS |  |  | ** |  |  | ** |  |
| C × N | NS |  |  | NS |  |  | NS |  |  | NS |  |  | NS |  |

^a^See Additional file 1 for experimental details.

^b^LYPJ, Liangyoupeijiu; YLY1, Y-liangyou 1; LY9348, HHZ, Huanghuazhan; YXYZ, Yuxiangyouzhan; Luoyou 9348; WY308, Wuyou 308; YLY2, Y-liangyou 2; YLY900, Y-liangyou 900; CY1000, Chaoyou 1000.

** represents significance at the 0.01 probability level; * represents significance at the 0.05 probability level; NS denotes non-significance.
