# Supplementary material for: Higher yields of hybrid rice do not depend on nitrogen fertilization under moderate to high soil fertility conditions
Source: Rice (N Y). 2017 Sep 21;10:43. doi: 10.1186/s12284-017-0182-1 (PMC5608657; doi:10.1186/s12284-017-0182-1)
Supplement: Supplementary file 3 — Table S2.Indigenous soil N uptake and labeled-N uptake in rice cultivars in a micro-plot experiment with application of 15 N labeled urea in Changsha, Hunan Province in China in 2013. (DOC 31 kb) [file 12284_2017_182_MOESM3_ESM.doc]

**Table S2.** Indigenous soil N uptake and labeled-N uptake in rice cultivars in a micro-plot experiment with application of ^15^N labeled urea in Changsha, Hunan Province in China in 2013^a^

| Cultivar^b^ | Indigenous soil N uptake (g m^−2^) | Labeled-N uptake (g m^−2^) |
| --- | --- | --- |
| LYPJ | 14.34 ab | 4.80 a |
| YLY1 | 14.47 a | 4.97 a |
| HHZ | 13.86 bc | 4.98 a |
| YXYZ | 13.62 c | 4.93 a |

Within a column, data with the same letters are not significantly different at the 0.05 probability level according to LSD test.

^a^See Additional file 1 for experimental details.

^b^LYPJ, Liangyoupeijiu; YLY1, Y-liangyou 1; HHZ, Huanghuazhan; YXYZ, Yuxiangyouzhan.
